# Biogeography and systematics of endemic island damselflies: The *Nesobasis* and *Melanesobasis* (Odonata: Zygoptera) of Fiji

**DOI:** 10.1002/ece3.3175

**Published:** 2017-08-18

**Authors:** Christopher D. Beatty, Melissa Sánchez Herrera, Jeffrey H. Skevington, Arash Rashed, Hans Van Gossum, Scott Kelso, Thomas N. Sherratt

**Affiliations:** ^1^ Department of Ecology & Evolutionary Biology Cornell University Ithaca NY USA; ^2^ Department of Biological Sciences Rutgers University Newark NJ USA; ^3^ Biology Program Faculty of Natural Sciences and Mathemathics Universidad del Rosario Bogotá Colombia; ^4^ Agriculture and Agri‐Food Canada Canadian National Collection of Insects Arachnids and Nematodes Ottawa ON Canada; ^5^ Department of Entomology, Plant Pathology and Nematology University of Idaho Aberdeen R & E Center Aberdeen ID USA; ^6^ Evolutionary Ecology Group University of Antwerp Antwerp Belgium; ^7^ Department of Biology Carleton University Ottawa ON Canada

**Keywords:** damselflies, Fiji Islands, male rarity, molecular clock, molecular phylogeny, oceanic islands, Odonata, sex ratio bias, Zygoptera

## Abstract

The study of island fauna has greatly informed our understanding of the evolution of diversity. We here examine the phylogenetics, biogeography, and diversification of the damselfly genera *Nesobasis* and *Melanesobasis*, endemic to the Fiji Islands, to explore mechanisms of speciation in these highly speciose groups. Using mitochondrial (COI, 12S) and nuclear (ITS) replicons, we recovered garli‐part maximum likelihood and mrbayes Bayesian phylogenetic hypotheses for 26 species of *Nesobasis* and eight species/subspecies of *Melanesobasis*. Biogeographical patterns were explored using lagrange and bayes‐lagrange and interpreted through beast relaxed clock dating analyses. We found that *Nesobasis* and *Melanesobasis* have radiated throughout Fiji, but are not sister groups. For *Nesobasis*, while the two largest islands of the archipelago—Viti Levu and Vanua Levu—currently host two distinct species assemblages, they do not represent phylogenetic clades; of the three major groupings each contains some Viti Levu and some Vanua Levu species, suggesting independent colonization events across the archipelago. Our beast analysis suggests a high level of species diversification around 2–6 Ma. Our ancestral area reconstruction (rasp‐lagrange) suggests that both dispersal and vicariance events contributed to the evolution of diversity. We thus conclude that the evolutionary history of *Nesobasis* and *Melanesobasis* is complex; while inter‐island dispersal followed by speciation (i.e., peripatry) has contributed to diversity, speciation within islands appears to have taken place a number of times as well. This speciation has taken place relatively recently and appears to be driven more by reproductive isolation than by ecological differentiation: while species in *Nesobasis* are morphologically distinct from one another, they are ecologically very similar, and currently are found to exist sympatrically throughout the islands on which they are distributed. We consider the potential for allopatric speciation within islands, as well as the influence of parasitic endosymbionts, to explain the high rates of speciation in these damselflies.

## 1 INTRODUCTION

Our understanding of speciation has been fundamentally influenced by the study of island fauna. From the writings of Darwin (1859) and Wallace (1855, 1880) to the transformative theories of insular zoology and biogeography of MacArthur and Wilson (1963, 1967) islands have served as model systems (Schoener, 2011; Warren et al., 2015) for the study of assembly rules, community dynamics, species radiations, and the evolution of diversity (Brewer, Carter, Croucher, & Gillespie, 2014; Lamichhaney et al., 2015). One of the key attributes that make islands unique places to study the evolution of diversity is their *relative* isolation; islands are not immediately connected to the mainland, and groups of islands have some isolation from one another, but occasional dispersal is possible, if limited. The nature of dispersal among islands is often associated with the mechanisms of island formation (Neall & Trewick, 2008); islands that were once part of the mainland will have potentially different patterns of biodiversity than islands formed de novo from volcanic activity; the relative ages of islands within an archipelago will also influence diversity patterns, as demonstrated by a variety of research projects in the Hawaiian islands (Brewer et al., 2014; Casquet et al., 2015; Jordan, Simon, & Polhemus, 2003; Wagner & Funk, 1995; Witter & Carr, 1988).

In these works, we see the influence of reproductive isolation; as groups of organisms colonize different islands, their physical isolation results in sufficient barriers to gene flow such that new species form. We also see the influence of niche differentiation, with new species forming on an island as they segregate into separate microhabitats, which partitions resources and minimizes competition (Hutchinson, 1959; Schoener, 1968). In some island chains, we see this as a repeated process, with some convergent evolution among species on different islands that have evolved in similar microhabitats (Brewer et al., 2014; Grant & Grant, 2008; Lack, 1947; Losos, Jackman, Larson, de Queiroz, & Rodriguez‐Schettin, 1998). If islands are sufficiently large, then speciation within the island (through both niche differentiation and allopatric speciation) can increase the predicted equilibrium species richness over that expected from immigration alone (Losos & Schluter, 2000).

We here present our investigations of two damselfly genera—the endemic *Nesobasis* and near‐endemic *Melanesobasis*, in the Fiji Islands in the South Pacific. *Nesobasis* consists of a large number of species (Donnelly, 1990); to date 21 species are described, with 15 more awaiting description (Donnelly, 1990; N. Donnelly pers. com. and own data). *Melanesobasis* includes a total of seven described species and one sub‐species; seven of these are found exclusively in Fiji (the eighth, *M. bicellulare*, is found on the island of Maewo in Vanuatu), and another two species are currently undescribed (Donnelly, 1984). *Nesobasis* represents one of the largest known radiations of endemic island odonates: only *Megalagrion* in the Hawaiian Islands has a comparable level of species diversity (Jordan, Simon, Foote, & Englund, 2005; Jordan et al., 2003; Polhemus, 1997).

These damselflies inhabit fast‐moving forested streams at medium to high elevations (100–750 m) (Beatty, van Gossum, & Sherratt, 2007; Donnelly, 1990; Van Gossum, Beatty, Tokota'a, & Sherratt, 2008). There is high morphological diversity among the species of *Nesobasis*, with large differences in coloration and size (see Fig. S1.1 in Appendix S1), as well as elaborate secondary reproductive structures in males and females (Beatty et al., 2007; Donnelly, 1990). Beyond this morphological diversity, it has also been demonstrated that some species within *Nesobasis* have highly female‐biased sex ratios at oviposition sites, with adult males being rare in many populations (Donnelly, 1994; Van Gossum et al., 2007).

**Figure 1:**
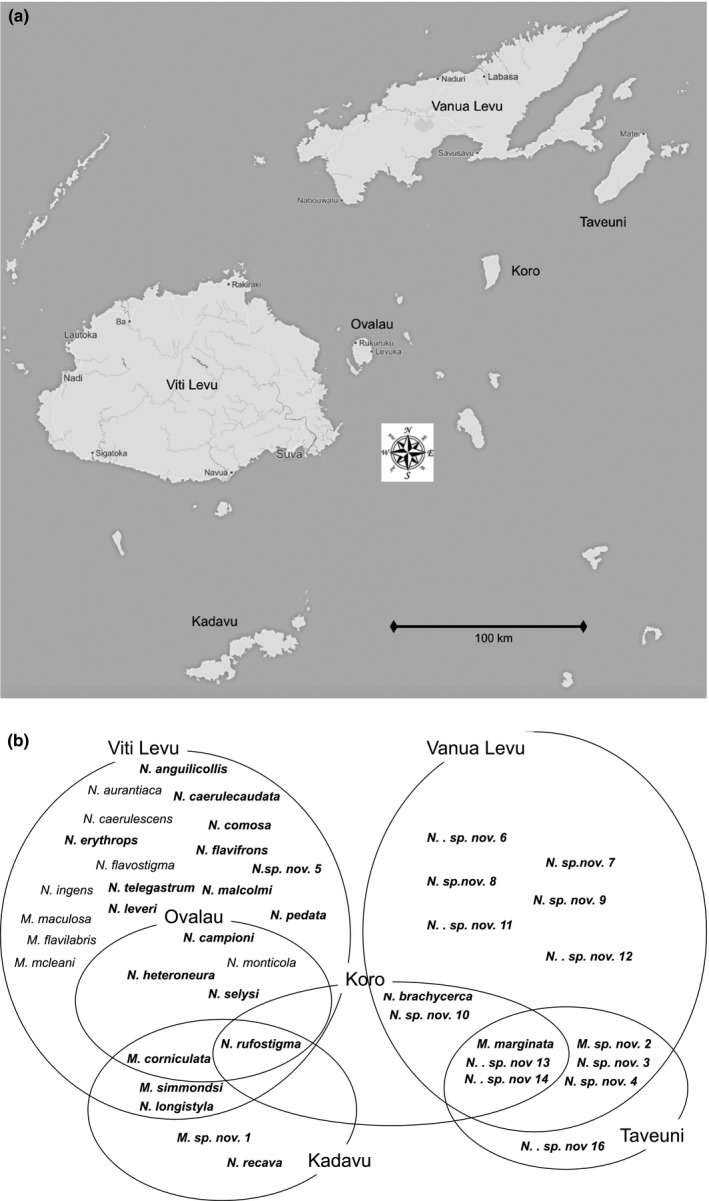
(a) Map of the central Fiji Islands, identifying the six islands where *Nesobasis* and *Melanesobasis* are currently known. (b) Venn diagram showing the distribution of *Nesobasis* and *Melanesobasis* species among the islands (adapted from Van Gossum et al., 2008). Here each island is represented as a circle; the species found on that island are within the circle, and overlapping circles occur where a species is found on more than one island. *Nesobasis* species assemblages form two distinct groups: One found on Viti Levu, Ovalau, and Kadavu, the other on Vanua Levu and Taveuni. Koro is the only island known to contain species from both assemblages

The geologic history of the Fiji islands is complex; the primary rocks of the islands are composed of intruded and extruded volcanics, uplifted marine sediments, and limestones (Rodda, 1994; Rodda & Kroenke, 1984). Data suggest that the first land formations in Fiji were island arc volcanics formed between 25 and 30 Ma; these are now found in the western part of Viti Levu, the largest of the Fiji islands (Figure 1a). These early portions of Viti Levu represented the easternmost extension of the Vitiaz Arc, a long chain that included the Solomons and Vanuatu (Hall, 2002). The formation of this arc system along with a 200–150 m drop in global sea levels during the Oligocene (30–28 Ma) is thought to have provided the earliest opportunities for eastward biotic migrations across the Vitiaz Arc (Haq, Hardenbol, & Vail, 1987). Shifts in plate movement have contributed to the expansion of Viti Levu to the south and east, with coral reef limestone accumulation expanding the island in the north. Expansion in the North Fiji Basin starting approximately 10 Ma resulted in the attachment of Viti Levu to the Pacific Plate; prior to 7 Ma Vanua Levu, the second largest island in the group, formed and rotated clockwise, with the entirety of Fiji rotating anti‐clockwise since that time. Taveuni—the third largest island, located just southeast of Vanua Levu—formed in the mid‐Quaternary with the eruption of over 150 vents (Neall & Trewick, 2008).

*Nesobasis* species are distributed over several islands within Fiji; there are two major assemblages of species, coinciding with the presence of two large islands in the archipelago: One assemblage is found on Viti Levu and its surrounding islands (Ovalau and Kadavu), the other on Vanua Levu and its surrounding islands (Taveuni and Koro) (Beatty et al., 2007; Donnelly, 1990; Van Gossum et al., 2008) with almost no overlap in spatial distribution between the two assemblages (Figure 1b). Knowing the distribution of *Nesobasis* and *Melanesobasis* within the Fiji Islands, and the developmental history of the islands, we make the following predictions about how speciation took place within and among these assemblages: (1) the *Nesobasis* found in Viti Levu will form three clades, that correspond to the three morphogroups (named ***comosa*** **,** ***erythrops,*** **and** ***longistyla*** after representative species) identified by Donnelly (1990) based on the diversification of secondary reproductive structures. We also predict that (2) the two assemblages of *Nesobasis*, associated with Viti Levu and Vanua Levu, respectively, represent two distinct clades within the genus, with isolation between the islands influencing the diversity within this group. We would make a similar prediction for the relationship between the *Melanesobasis* species on Viti Levu and Vanua Levu. Finally, we predict (3) that species with female‐biased sex ratios are likely related to one another, as this trait may reflect common ancestry.

To test these predictions, we first present phylogenetic hypotheses of species relationships, in the genera *Nesobasis* and *Melanesobasis*, based on molecular data obtained from both mitochondrial and nuclear sequences. We then estimate the diversification rate within these groups through a beast relaxed molecular clock and make predictions about dispersal‐mediated speciation (i.e., peripatric speciation), vicariance, and extinction within these groups using rasp‐lagrange. Finally, we map sex ratio data for *Nesobasis* onto our phylogeny to observe how species with female‐biased sex ratios are distributed within the phylogeny and among islands.

## 2 MATERIALS AND METHODS

### 2.1 Taxon sampling

We analyzed 15 of 21 described *Nesobasis* species, as well as eleven of 15 taxa that are currently being prepared for description. For *Melanesobasis,* six of eight described species/subspecies and two undescribed species were also obtained for analysis (the two outstanding species from *Melanesobasis*,* M. prolixa,* and *M. bicellulare* are found on islands quite distant from our study islands) (see Table S2.1 in Appendix S2 for specimen details). *Melanesobasis corniculata marginata* is currently described as a subspecies of *M. corniculata corniculata* (Donnelly, 1984). While we recognize this relationship, for the sake of simplicity we will refer to these as *M. corniculata and M. marginata* throughout the text. All known “female‐biased” species were included in the analysis. These include *N. comosa*,* N. heteroneura*,* N. malcolmi*,* N. rufostigma,* and *N. sp. nov. 9* (Van Gossum et al., 2007). Population‐level sampling was limited to five species (*N. anguilicollis*,* N. brachycerca, N. comosa, N. rufostigma,* and *N. selysi*), of which two species have specimens from multiple islands (*N. brachycerca* and *N. rufostigma*) (Table S2.1). Additional members of the family Coenagrionidae (*Pseudagrion ignifer* and *Ischnura heterosticta*) were included within the analyses. *Idiocnemis pruinescens*,* Idiocnemis louisiadensis*, and *Platycnemis acutipennis* (all members of family Platycnemicidae) were defined as outgroups for all analyses. Based on the most recent phylogenies of the Odonata and Zygoptera, Platycnemididae, Coenagrionidae, Pseudostigmatidae, and Protoneuridae encompass the Coenagrionoidea superfamily (Dijkstra, Kalkman, Dow, Stokvis, & Van Tol, 2014; Dijkstra et al., 2013). Adult damselflies were collected and preserved in 95% ethanol, transferred into fresh absolute alcohol twice after collection to remove most water from the specimens' tissues, then databased and placed in a −80°C freezer for storage until needed.

### 2.2 DNA extraction, amplification, sequencing, and alignment

Insect DNA was extracted using Qiagen QIAamp DNA mini kits following manufacturer protocols. Specimens were sequenced for COI (~1,420 bp), 12S (~331 bp) and ITS (~837 bp) (see Table S2.2 in Appendix S2 for primer details). All PCR reactions were performed on an Eppendorf ep gradient S Mastercycler (Eppendorf AG, Hamburg, Germany). PCR amplifications were performed in a total volume of 50 μl, containing 0.625 mmol/L MgCl_2_, 0.4 μmol/L each primer, 0.8 mmol/L dNTP mixture, 5 μl Ex Taq Polymerase reaction buffer (containing 20 mmol/L MgCl_2_), 1.25 units of Ex Taq HS DNA Polymerase (Takara Bio USA, Madison, WI, USA), 33.5 μl dH2O, and 2 μl gDNA. All cycling started with a 3‐min hot start at 94°C followed by: 12S: 35 cycles of 94°C for 1 min, 54°C for 1 min, and 72°C for 2 min; COI (3′ region): 39 cycles of 94°C for 50 s, 58°C for 50 s, and 72°C for 1 min; COI (5′ region): 39 cycles of 94°C for 1 min, 45–50°C for 1 min, and 72°C for 2 min; ribosomal spacers, ITS1 and ITS2, and ribosomal 5.8S: 30 cycles of 95°C for 1 min, 52°C for 90 s, and 72°C for 2 min. Amplification products were purified with a Qiagen PCR Purification Kit (Qiagen Inc., Mississauga, ON, Canada), following manufacturer protocols.

DNA sequencing reactions were performed at the Agriculture & Agri‐Food Canada Core Sequencing Facility (Ottawa, Ontario, Canada) in a total volume of 10 μl, using an ABI BigDye Terminator v3.1 Cycle Sequencing Kit (PE Applied Biosystems, Foster City, CA, USA) following manufacturer protocols. Sequencing reactions were purified using the ABI Ethanol/EDTA/Sodium Acetate Precipitation protocol. Purified sequencing reactions were analyzed on an ABI 3130 xl Genetic Analyzer (PE Applied Biosystems). Contigs and chromatogram examination were made using Sequencher 5.2.4 (Gene Codes Corp., Ann Arbor, MI, USA). Alignment of COI was performed manually and checked to ensure that there were no stop codons or frame shifts. Alignment was straightforward, and there were no indels. We employed clustalX to generate separate alignments for 12S and ITS, using default parameters. A total evidence matrix was assembled in MacClade 4.08 (Maddison & Maddison, 2001) using our hand‐aligned COI and clustal‐aligned 12S and ITS (GenBank accession numbers for all resulting sequences can be found in Table S2.3, Appendix S2).

### 2.3 Phylogenetic methods

Phylogenetic relationships among the taxa were reconstructed using three different criteria: maximum parsimony (MP), maximum likelihood (ML), and Bayesian inference (BI). Independent analyses of each replicon (COI, 12S, and ITS), as well as total evidence using partitions, were reconstructed for each criterion. garli‐part 2.0 (Zwickl, 2006) was used for ML, mrbayes 3.2.1 (Huelsenbeck & Ronquist, 2002) for BI and TNT (Goloboff, Farris, & Nixon, 2000) for MP. For each replicon, the substitution model was obtained using both the Akaike Information Criterion (AIC) and Bayesian Information Criterion (BIC) in jmodeltest2 (Posada, 2008). The selected model for the COI and ITS replicons was TPM2uf + I + G; for 12S, the model was HKY + I + G. We used the selected models in our ML independent and combined partition analyses. The supports for the branches were estimated from a total of 1,000 bootstrap pseudoreplicates. The consensus trees were summarized using sumtrees (Sukumaran & Holder, 2010). For our BI, we used the GTR + I + G model for COI and ITS due to mrbayes constraints. Four different heated MCMC chains were used; we ran 1 × 10^7^ generations sampling every 100 cycles. Multiples runs (~4) were performed to ensure convergence of the posterior distributions assessed using tracer v. 1.5 (Rambaut & Drummond, 2007). Majority rule 50% posterior probability trees were obtained as consensus final topologies after burning 25% of the generations. The MP analyses were performed using a traditional heuristic search under the subtree‐pruning‐regrafting branch swapping algorithm and random addition of taxa. All multistate characters were treated as nonadditive. Polymorphisms and gaps were treated as missing data. Strict and major rule consensuses and important statistics—tree length (TL), consistency index (CI), and retention index (RI)—were obtained. Finally, the branch supports were obtained using 1,000 bootstrap psuedoreplicates using the same searching options (i.e., heuristic search, SPR). All ML and BI trees were visualized using figtree v. 1.4 (Rambaut, 2010), while for MP, we used treeview (Saldanha, 2004).

### 2.4 Divergence time estimation analyses

We used our partitioned dataset to run relaxed clock molecular dating analyses using beast v1.8 (Drummond, Suchard, Xie, & Rambaut, 2012). The three partitions (COI, 12S, and ITS), clock, and site models were unlinked. We implemented the GTR + G + I model for COI and ITS and HKY + G + I for 12S. A random starting tree was used in the analysis, and biogeographical features such as island emergences (3) and fossils (1) were used as calibration points (see Table S2.4 in Appendix S2). Despite the fact that biogeographical events may not be reliable calibration points (Parham et al., 2012), our volcanic island system allowed us to extrapolate the emergence ages as calibration points, using normal distributions as the prior distribution probabilities. We ran four independent runs for 10 million generations to ensure convergence of the MCMC; these were checked using tracer 1.4 (Rambaut & Drummond, 2007). Finally, all the runs were combined using logcombiner v 1.8 (Drummond et al., 2012). The dated ultrametric tree was obtained using treeannotator v 1.8 (Huelsenbeck & Ronquist, 2002) and visualized using figtree v. 1.4 (Rambaut, 2010). To determine rates of diversification throughout the tree, we implemented the Generalized Mixed Yule Coalescent (GMYC) likelihood method for delimiting species by fitting within‐ and between‐species branching models to reconstructed gene trees (Fujisawa & Barraclough, 2013). The GMYC model assumes that species are monophyletic. It relies on a single, or multiple, thresholds to delimit species nodes defining the most common ancestor of the species. The “threshold time” estimates differences between inter‐ (i.e., diversification) and intraspecific (i.e., coalescence) branching events. This model requires an ultrametric tree, so we used the COI partition tree obtained in BEAST.

### 2.5 Biogeographical analyses

To estimate the ancestral ranges of *Nesobasis* and *Melanesobasis,* we ran the Dispersal‐Extinction‐Cladogenesis (DEC) model using lagrange (Ree, Moore, Webb, & Donoghue, 2005) and bayes‐ lagrange (S‐DEC, Smith , 2009) under the Reconstructing Ancestral State in Phylogenies (rasp) v. 3.0 platform (Yu et al. 2015). Our dated beast ultrametric tree was use to root the age to 39 Ma. We assigned the taxa with one or more of the following areas: Viti Levu (A), Vanua Levu (B), Kadavu (C), Taveuni (D), Ovalau (E), and Koro (F) (outgroups were designated as Mainland (G)). We used the following time dispersal constraints: (0) possible in all seven areas, (1) 7 Ma dispersal between Mainland, Viti Levu, and Vanua Levu, (2) 12 Ma dispersal between Mainland and Viti Levu, and (3) 39 Ma only Mainland. The taxa ranges are based on the published collection localities (Beatty et al., 2007; Donnelly, 1984, 1990; Van Gossum et al., 2008). We set several dispersal ability constraints based on the island emergence ages (see Table S2.5 in Appendix S2).

## 3 RESULTS

### 3.1 Phylogenetic reconstruction

**Figure 2:**
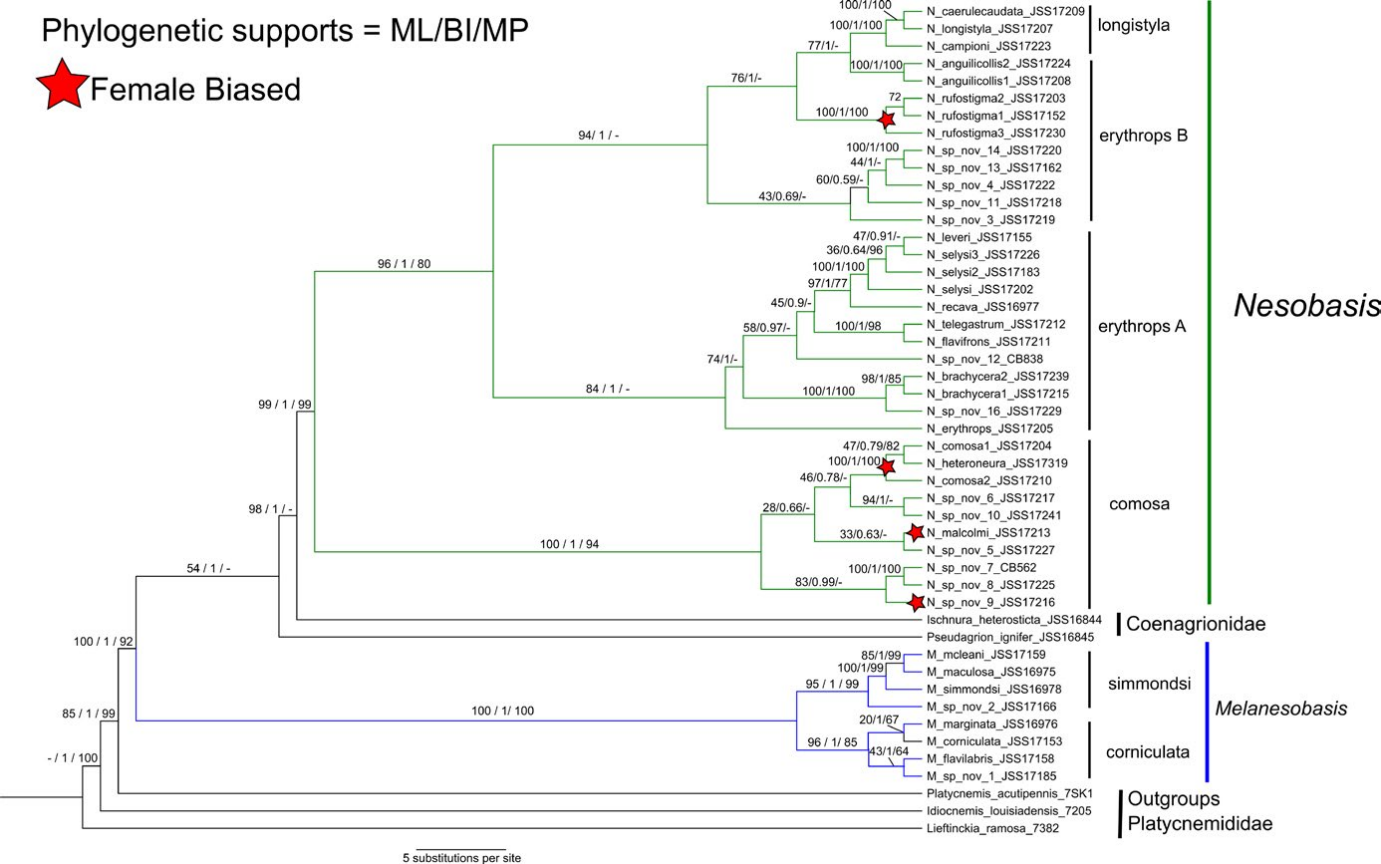
Combined phylogenetic tree of *Nesobasis* and *Melanesobasis* species. Phylogenetic supports for branches within the tree are shown for each phylogenetic method employed (maximum likelihood (ML), Bayesian inference (BI), and maximum parsimony (MP). Species that have female‐biased populations are indicated by a star

The phylogenetic relationships recovered for the total evidence analyses (Figure 2) and the independent replicons—COI, 12S, and ITS (Figs. S1.2 and S1.3 in Appendix S1)—are highly congruent among the three criteria (ML, BI and MP). Only a few differences in the support values throughout the analyses were observed. The monophyly of both genera—*Nesobasis* and *Melanesobasis*—was recovered and highly supported (*Nesobasis* has a 99% bootstrap for ML and MP inference and a posterior probability of 1 for the BI; *Melanesobasis* has a 100% for ML and MP and a posterior probability of 1). However, our topology suggests that these two genera are not sister clades; *Melanesobasis* was supported as sister to all coenagrionids, while *Nesobasis* is more closely related to *Ischnura* and *Pseudagrion* (Fig. 2). Two highly supported sister reciprocal clades show the relationships within *Melanesobasis*; one hereafter called the ***corniculata*** **clade**, including *M. marginata, M. corniculata, M. flavilabris* and *M. sp. nov.1* and the second hereafter called the ***simmondsi*** **clade**, which encompasses *M. simmondsi*,* M*. mcleani, **M.** maculosa, and *M*.*sp. nov*.***2***, (Figure 2). *Nesobasis* is divided into three distinct clades; however, the position of this genus within the family Coenagrionidae still needs to be thoroughly tested. *Nesobasis comosa*,* N. heteroneura, N. malcomi,* and *N. sp.nov. 5*—all from Viti Levu—along with*, N. sp.nov. 6, N. sp .nov. 7&8* (putatively a female and male of the same species), *N. sp.nov. 9,* and *N. sp.nov. 10* from Vanua Levu comprise a highly supported sister clade (hereafter called the ***comosa*** **clade**) to all other *Nesobasis* (Figure 2). The remaining species were grouped into two supported sister clades which encompass the morphologically defined ***erythrops*** and ***longistyla*** groups (Donnelly, 1990). The first of these clades includes the ***erythrops*** species *N. erythrops*,* N. leveri*,* N. selysi, N. telegastrum, N. flavifrons* (all from Viti Levu)*, N. recava* (endemic to Kadavu)*, N. sp. nov. 12, N. brachycera* (one each from Vanua Levu and Koro), *and N. sp.nov. 16* (Taveuni) (see clade erythrops A, Figure 2). The other clade includes the ***erythrops*** species *N. anguilicollis and N. rufostigma* (Viti Levu)*, N. sp. nov. 3, N. sp. nov. 4*,* N. sp. nov. 11 (all from Vanua Levu),* and *N. sp. nov. 13 & 14* (possibly the same species, but collected on Taveuni and Vanua Levu, respectively); this clade also includes a lineage containing the ***longistyla*** species *N. logistyla, N. caerulecaudata, and N. campioni,* (See clade erythrops B, Figure 2). The ***longistyla*** species group as defined by Donnelly (1990) renders the ***erythrops*** group paraphyletic in our analyses.

Species with “female‐biased” adult sex ratios appear at four independent localities within the tree (Figure 2, indicated with a star); three of these are within the ***comosa*** group, while one, *N. rufostigma*, is in the ***erythrops*** group, clade B.

### 3.2 Divergence time analysis

**Figure 3:**
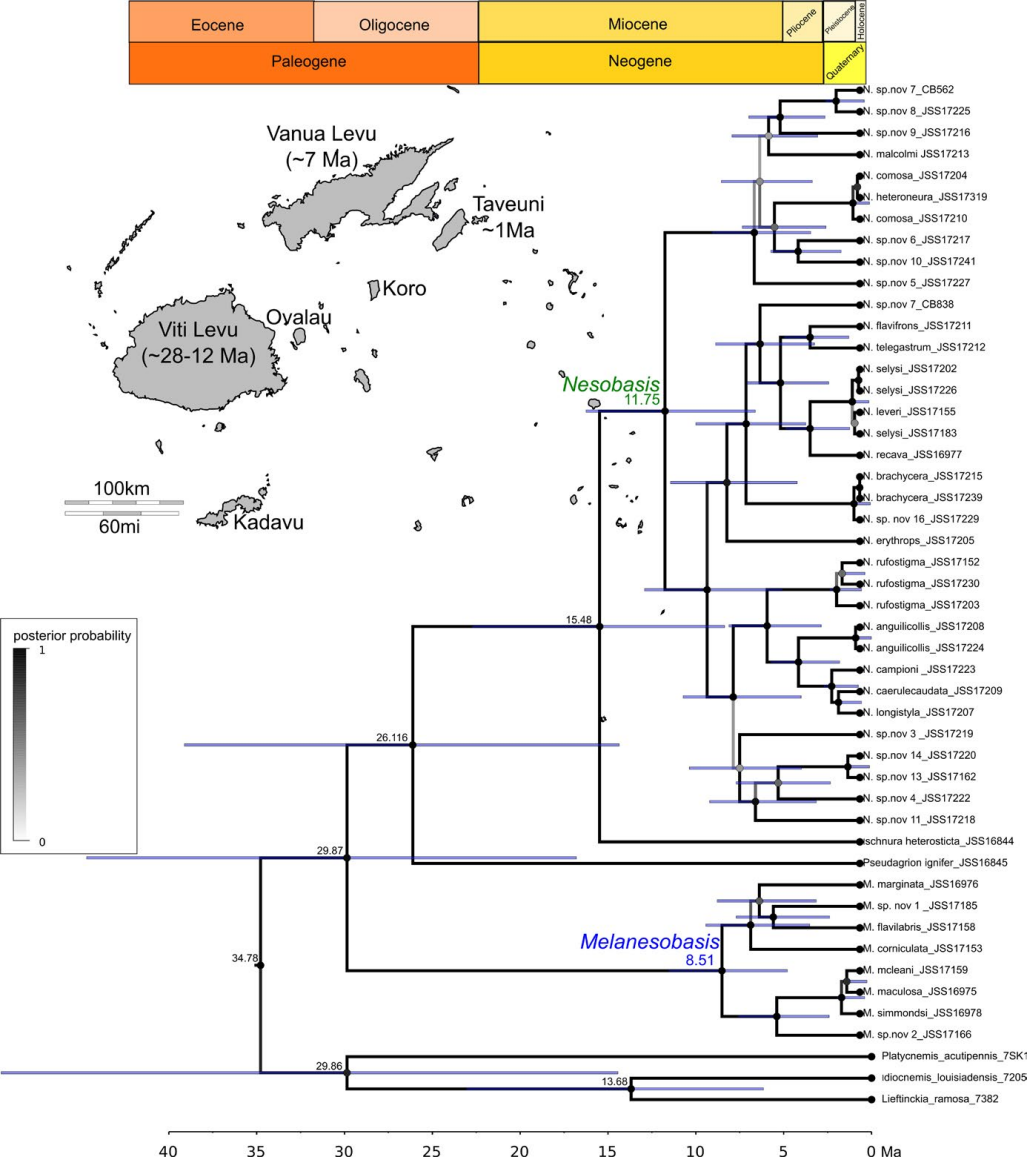
BEAST relaxed clock showing estimations of timing of each node with error

**Figure 4:**
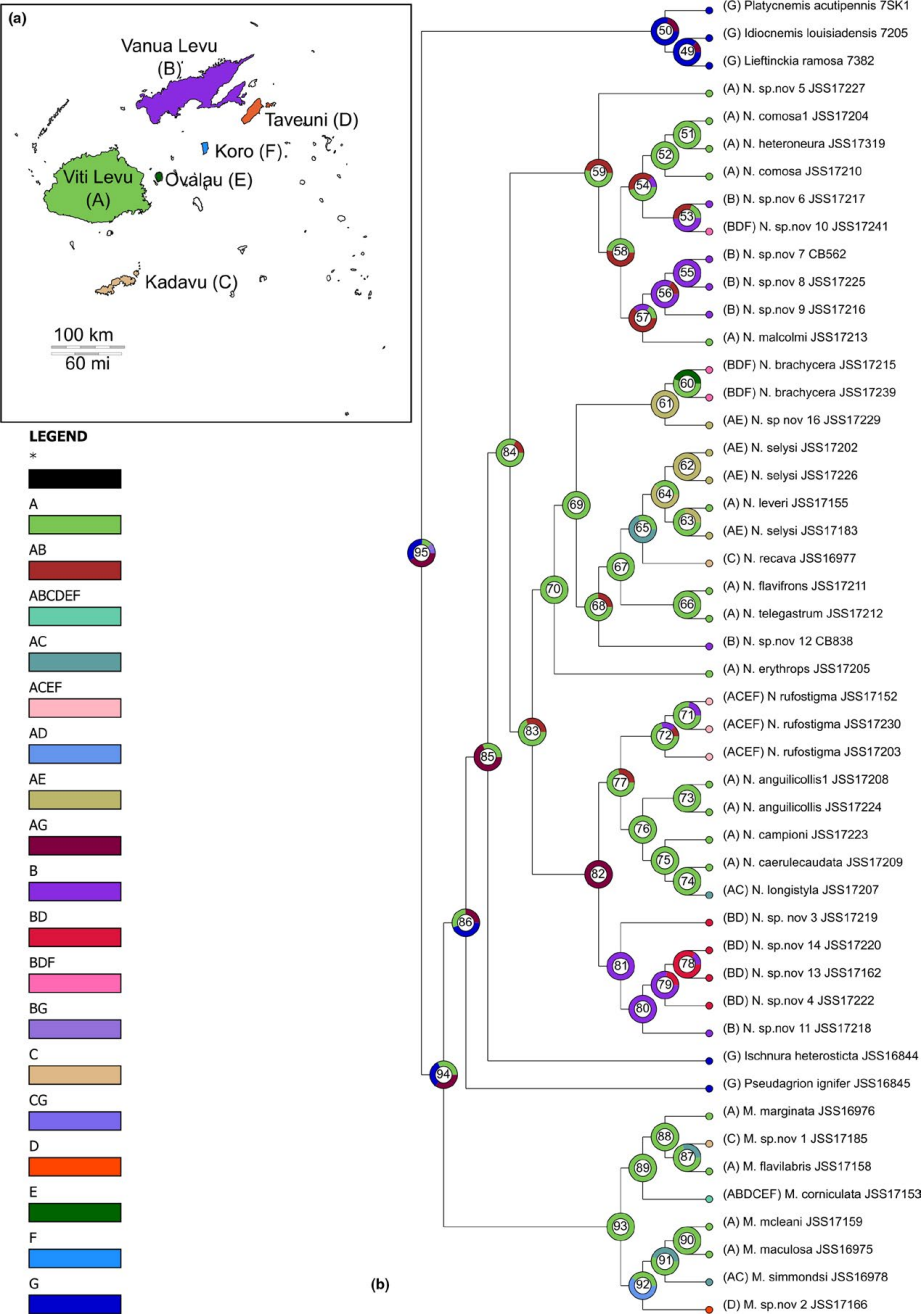
LaGrange extinction‐vicariance analysis

Our beast divergence time calibrated topology suggests that the common ancestor to all the coenagrionids included in this analysis diverged sometime around the early Oligocene ~30 Ma. Furthermore, it suggests that the two major clades recovered within *Melanesobasis* diverged around ~8 (CI = 5–12) Ma during the late Miocene (Figure 3). *Melanesobasis corniculata* seems to be the oldest extant species at ~6 Ma, while *M. mcleani* and *M. maculosa* seem to be the youngest with a shared common ancestor only ~730,000 years ago during the Late Pleistocene. Our results for *Nesobasis* suggest that they shared a common ancestor with the species *Ischnura heterosticta,* diverging ~16 Ma, and the diversification of the genus possibly started ~12 (CI = 7–16) Ma during the middle Miocene. The clades containing the ***longistyla*** and ***erythrops*** groups diverged from each other around 8 Ma, almost parallel to the *Melanesobasis* diversification. Our topology also suggests that these clades had a parallel diversification across the Fiji islands; however, some of the ***erythrops*** species (including *N.selysi*,* N.leveri*,* N. sp.nov* 16, and *N. brachycera*) show very recent diversification dates around 330,000 to 10,000 years ago. The ***comosa*** clade also started its diversification parallel to *Melanesobasis* during the late Miocene ~6 Ma. The oldest species within this clade is *N. sp. nov. 5* and the youngest are *N. comosa* and *N. heteroneura* ~390,000–141,000 years ago. Finally, the latter results suggest a peak of diversification for most of the extant species during the Pleistocene and Holocene epochs. The GMYC model of our COI gene tree revealed three distinct thresholds where the branching rates change within our *Nesobasis* + *Melanesobasis* phylogeny: the first at ~8 Ma, the second at ~6.6 Ma, and the third and most recent at 1.2 Ma (Figure 4).

### 3.3 Biogeographical patterns

**Figure 5:**
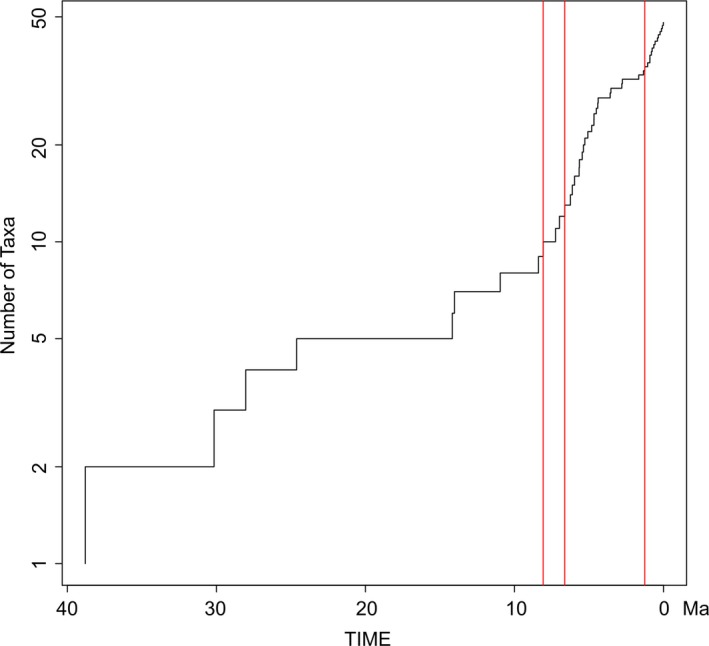
Generalized Mixed Yule Coalescent (GMYC) showing rates of diversification through time in our *Nesobasis* and *Melanesobasis* phylogeny. Vertical lines indicate points of significant increases in diversification rate

Our estimated DEC and S‐DEC models were consistent overall; however, there were a few more supported dispersal and extinction events in the S‐DEC model (see Table S2.5 in Appendix S2). The DEC model estimated 50 dispersal, 10 vicariance, and 1‐extinction events, while the S‐DEC model estimated 52, 10, and 2 events, respectively (Figure 5). Both analyses support a high dispersal from Viti Levu to the other islands (DEC 32 events and S‐DEC 31 events) and relatively high speciation within Viti Levu as well (26 speciation events for both models, Table S2.5 Appendix S2). For both *Melanesobasis* (Figure 5, Node 93, 100%|DEC & 97.52%|S‐DEC) and *Nesobasis* (Node 84, 82.23%|DEC & 75.84%|S‐DEC), our analyses support with a high probability Viti Levu as their ancestral area. We recovered *Melanesobasis* mainly as Viti Levu taxa with a few species with high dispersal abilities. Within the ***corniculata*** clade, there are five highly supported (Figure 5, Node 89, 1 for DEC and S‐DEC) dispersal events due to the presence of *M. corniculata* in almost all of the islands under study. Furthermore, for node 87 our analyses support two dispersal events and one vicariance event due to the presence of *M. sp.nov 1* only in Kadavu. The ***simmondsi*** clade shows a strange vicariance route with a low support to the island of Taveuni due to *M. sp. nov.2* (Figure 5, Node 92, 0.3335|DEC & 0.3347|S‐DEC).

The three distinct *Nesobasis* clades show interesting patterns. First, the ***comosa*** clade shows a 50/50 split between Viti Levu and Viti Levu/Vanua Levu as the ancestral area (Figure 5, Node 59). In this clade, there is at least one vicariance event between Viti Levu and Vanua Levu that yields two independent colonization events to Vanua Levu around ~5 Ma (Nodes 54, 57: Figure 5). For the clades that include the ***erythrops*** and ***longistyla*** groups, our analyses support Viti Levu (Figure 5, Node 83, 67.28%|DEC & 60.84%|S‐DEC) or Viti Levu/Vanua Levu (Figure 5, Node 83, 32.72%|DEC & 38.70%|S‐DEC) as their ancestral area. The first ***erythrops*** clade supports multiple independent dispersals to several of the islands. The first colonization event to Vanua Levu was consistent with the same vicariance event around ~5 Ma (Figure 5, Node 68, 0.7433|DEC & 0.6979|S‐DEC) supported by the presence of *N. sp. nov. 12* in Vanua Levu. The second colonization to Vanua Levu was more recent ~10,800 years ago supported by *N. brachycera*, which is also consistent with a possible extinction of this species in Viti Levu (Figure 5, Node 60, 0.5563|DEC & 0.5625|S‐DEC). For the second clade containing both ***erythrops*** and ***longistyla*** species, Viti Levu and Vanua Levu (Figure 5, Node 80, 100%|DEC & 100%|S‐DEC) are highly supported as the ancestral areas. Our models suggest that there was only one colonization of Vanua Levu that happened around 6.8 Ma (Figure 5, Node 81, 1 for both models) by the species *N. sp. nov. 3,* which possibly then dispersed to Taveuni. The remaining species within this clade are mainly dispersed across Viti Levu and its nearest islands of Kadavu and Ovalau, although one species, *N. rufostigma*, has also dispersed to Koro (Figure 5, Node 77, 0.553|DEC and 0.4761|S‐DEC). Overall, for *genus Nesobasis,* there appear to be four independent colonizations from Viti Levu to Vanua Levu.

## 4 DISCUSSION

### 4.1 Species concepts in *Nesobasis* and *Melanesobasis*

Our results generally support our first prediction, as the Viti Levu species groups suggested by Donnelly (1990) are mainly in agreement with the structure of our tree; one clade represents the ***comosa*** group (Figure 2), while another clade contains Viti Levu species in the ***erythrops*** group (erythrops A); the third clade contains species belonging to the ***longistyla*** group (a group of species defined by the shared trait of vestigial paraprocts in the male; these species do appear as a distinct clade within the phylogeny), while the remaining species are part of the ***erythrops*** group (erythrops B). Thus, the ***erythrops*** group *sensu stricto* could be considered paraphyletic. The species in the two ***erythrops*** clades do not differ in any notable way based on their taxonomy. The phylogeny also identifies Vanua Levu species that are members of each of the three clades described above, although these were not included in Donnelly's original paper.

For *Melanesobasis*, while predominantly found on Viti Levu, the relationship of *M. corniculata* and *M. marginata* reflects the similarities between these taxa as identified by Donnelly (1984), prompting him to describe *M. marginata* as a subspecies of *M. corniculata*. The ***simmondsi*** clade includes the namesake species as well as two others, *M. maculosa* and *M. mcleani*, which Donnelly suggested to all be closely related, *M. maculosa* being smaller and paler, while *M. mcleani* is larger and darker, with anal appendages more similar to those in *M. corniculata*.

The relative positions of *Nesobasis* and *Melanesobasis* in our phylogeny offers some interesting light on a persistent question concerning the relationship between these groups and their positions in the families of Coenagrionidae and Platycnemididae. Donnelly (1984), in erecting *Melanesobasis* and moving some species previously attributed to *Nesobasis* into it, pointed out several shared morphological traits between the groups, including wing venation patterns and the presence of tarsal claws, which suggest a close relationship between these two genera. *Melanesobasis* is distinct from *Nesobasis* due to its large inferior appendages in the male, its overall darker coloration, and its generally more dense wing venation.

*Melanesobasis* also shows similarities to species in the genus *Lieftinckia* in the family Platycnemididae, such as undulant wing margins (a trait seen to a lesser extent in some species of *Nesobasis*) long legs with long setae, and a relatively wide head and stout thorax. Donnelly suggested that these traits confused the position of *Melanesobasis*, making it unclear whether this genus might fall in either Coenagrionidae or Platycnemididae (Donnelly, 1984).

Our current analysis suggests that combining *Melanesobasis* with the three genera of Platycnemididae we used as outgroups would create a paraphyletic group, thus making its position in this family unlikely. *Melanesobasis* is basal to all of the other Coenagrionidae in our tree; this suggests that the genus could be assigned to Coenagrionidae, but as a relatively ancestral component of that family. Alternatively, *Melanesobasis* could be assigned to its own monophyletic family, intermediate between Platycnemididae and Coenagrionidae.

### 4.2 *Biogeography of* Nesobasis *and* Melanesobasis

The extant species of *Nesobasis* are divided into two assemblages geographically—one associated with Viti Levu and its surrounding islands (Ovalau, which contains six species that are all found on Viti Levu, and the larger and more‐distant Kadavu, which contains two species from Viti Levu and a single endemic, *N. recava* (Figure 1b)) and the other with Vanua Levu and its neighboring island of Taveuni (Figure 1b). Thus, the distributions of the two groups are quite separate from one another: The only exception is the small island of Koro located mid‐way between the two large islands, which has *N. rufostigma*, a “Viti Levu” species, but otherwise hosts only “Vanua Levu” species (Figure 1b). Our tree, however, shows a more complex relationship: each of the major clades of *Nesobasis* contains both Viti Levu and Vanua Levu species. In some cases, a single Vanua Levu species is found within a Viti Levu clade, while in at least two incidences, multiple Vanua Levu species are clustered together. Thus, our second prediction, that the Viti Levu and Vanua Levu species would form distinct clades, is not supported. *Melanesobasis* shows a different pattern, with the majority of the species found on Viti Levu; one of these, *M. corniculata*, is also found on Ovalau, while Kadavu hosts *M. simmondsi* and the endemic *M. sp. nov. 1*. The Vanua Levu group hosts two separate species /subspecies:, *M. sp. nov. 2* is found on Vanua Levu and Tavenui; *M. marginata*, a subspecies of *M. corniculata*, is found on these two islands as well as Koro (Figure 1b). Thus, the ***simmondsi*** and ***corniculata*** clades have each contributed a species/subspecies to the Vanua Levu group (Figure 2).

### 4.3 Divergence times in *Nesobasis* and *Melanesobasis*

Our estimated timeline for the development of diversity suggests an increased diversification rate from 2 to 6 Ma, with a number of species forming more recently, in the Pleistocene and Holocene. The number and size of islands available to *Nesobasis* and *Melanesobasis* began to increase through this time, with Viti Levu and Vanua Levu accruing more landmass, and other islands such as Kadavu and Taveuni beginning to form (Neall & Trewick, 2008). The biogeographical patterns identified in our analysis suggest that a mix of dispersal and vicariance contributed to the overall diversity within our two study genera (Figure 4). In *Melanesobasis*, of the eight species/subspecies studied, five appear to have emerged on Viti Levu, while three (*M. marginata*,* M. sp. nov.1*,* M. sp. nov. 2*) are the results of dispersal events between islands. Four species, two from Viti Levu and two from Vanua Levu, subsequently expanded their distribution to other islands. For *Nesobasis*, a total of 14 species appear to have developed on Viti Levu. Six dispersal events have resulted in new species: Three of these are single‐species events (*N. brachycerca* and *N. sp. nov. 12* onto Vanua Levu, *N. recava* onto Kadavu), while we have identified three other points within the tree that appear to be dispersal events (nodes 73, 78, and 83 in our rasp‐lagrange analysis, Figure 5), resulting in multiple species on Vanua Levu. One of these speciation events, for *N. brachycerca*, is predicted from our results to have been associated with a subsequent extinction on Viti Levu. Including these diversifications, we estimate that around 20 of our analyzed *Nesobasis* speciated within a single island, while at most, six are the result of dispersal. Thus, within *Nesobasis*, 73% of species resulted from within‐island diversification, while 63% of *Melanesobasis* formed in this way. Our most parsimonious interpretation of our rasp‐lagrange results suggests that movement between the island groups was predominantly represented by movement from Viti Levu to Vanua Levu, which is in line with the relative ages of the islands.

### 4.4 Drivers of speciation in *Nesobasis* and *Melanesobasis*

Using a metacommunity simulation modeling approach, McPeek (2007, 2008) explored the relative influence of ecological mechanisms in speciation (the “Hutchisonian” model of niche differentiation and species coexistence (Hutchinson, 1959)) versus speciation that results in little ecological diversification in new species, such as through sexual selection. When the resulting species lineages were studied, clades showing decelerating lineage accumulation rates (those that diversify early in their history) were those that had diversified by ecological modes of speciation, whereas clades showing accelerating lineage accumulation rates (relatively recent increases in speciation over evolutionary time) are those that had diversified primarily by modes of speciation that generate little or no ecological diversification (McPeek, 2008).

Looking specifically at diversity within islands, Whittaker and colleagues (Whittaker et al. 2008) developed a general dynamic model (GDM) of ocean island biogeography to provide an explanation of biodiversity patterns based on fundamental biogeographical processes—speciation, immigration, extinction—through time and in relation to island ontogeny. This work incorporates the fundamentals of the Island Biogeography Theory of MacArthur and Wilson (1963, 1967) as well as a relationship between island age and diversity, and rates of endemism as a function of island size and isolation (Heaney, 2000). The predictions of this model for isolated archipelagos like Fiji would be for relatively high rates of cladogenesis leading to endemic species, through niche differentiation or allopatry, as the island goes through its formative stage (increasing in size, elevation, and habitat complexity). This species diversity should peak relatively early in the life of the island, prior to island subsidence and erosion (Whittaker, et al. 2008).

The results of our GMYC model suggest relatively recent diversification within these genera: While *Nesobasis* and *Melanesobasis* are estimated to have originated 11 and 8 Ma, respectively, diversification rates within these groups have increased relatively recently, with major increases at 6 and 1.2 Ma. While some of these speciation events are associated with movements to more recently formed islands, the majority took place within one of the two largest islands, Viti Levu or Vanua Levu. These results appear to be in line with McPeek's (2008) suggestions for nonecological species diversification and differ somewhat from the predictions of the GDM, which would predict higher rates of cladogenesis at an earlier point in the formation of these islands, associated with differentiation into empty niche space on the islands. While it is difficult to estimate the rate at which new niches would have appeared in the original formation of these islands, increased rates of speciation on Viti Levu and Vanua Levu appear well after the earliest estimated time of formation of these islands; thus, Viti Levu and Vanua Levu were not newly arisen when *Nesobasis* and *Melanesobasis* began to diversify. If these islands were well‐developed, there would likely already have been a number of niches available; thus, our phylogenetic results generally concur with predictions for nonecological diversification.

Based on previous research, we do not see major ecological diversification within the *Nesobasis* and *Melanesobasis* damselflies; while some species in *Nesobasis* are found more commonly at higher elevation sites (Beatty et al., 2007; Donnelly, 1990), the majority of species in the genus are found sympatrically in a number of streams and small rivers; total *Nesobasis* species diversity has been found to range from one species to as many as 12 species at a single site (Beatty et al., 2007; Donnelly, 1990; Van Gossum et al., 2008). These species appear to use the same larval and adult habitats, based on larval sampling in a few streams (CDB, unpublished data). Two species of *Nesobasis*,* N. ingens* on Viti Levu (not included in our analysis as fresh specimens were unavailable for DNA extraction) and *N. sp, nov. 8* on Vanua Levu, are larger and longer‐bodied than most other members of the genus, more resembling species of damselfly that oviposit in tree holes or epiphytes (Silsby, 2001, pp.123–124). The larvae of these species have not been collected, and so it is possible that they have diversified in their oviposition habitat, but the majority of *Nesobasis* species oviposit within the channels of small streams in Fiji.

While there is very little ecological diversification within these species, there is significant morphological diversity, especially within *Nesobasis*. These species differ greatly in coloration, both within and among clades, and there is also significant diversity in the structures involved in the attachment of males and females during mating, structures that tend to function as “lock and key” mechanisms in copula (Donnelly, 1990). In some species of *Megalagrion*, it has been shown that color variation in sexual dimorphism is associated with elevational distribution within islands, suggesting that this color is an adaptive response to increasing exposure to UV radiation (Cooper, Brown, & Getty, 2016). More generally, coloration is a trait used by odonates in mate selection (Battin, 1993; Tynkkynen, Kotiaho, & Svensson, 2008).

Another possible source of diversity is that island size and habitat patchiness may drive speciation, especially with larger islands facilitating allopatric speciation, with species originating in different, isolated parts of the island (in different valleys for example) (Losos, 1996; Losos & Schluter, 2000; Heaney, 2000; Whittaker et al. 2008). with subsequent range expansions. It is worth noting that most of the species in *Nesobasis* and *Melanesobasis* are currently found throughout the islands they inhabit, such that species do not show significant distributional differences within an island. While different odonate species display different potentials for dispersal, movement between ponds (Conrad, Willson, Harvey, Thomas, & Sherratt, 1999; Geenen, Jordaens, De Block, Stoks, & De Bruyn, 2000) and through stream networks (Chaput‐Bardy, Lemaire, Picard, & Secondi, 2008) is not uncommon. If their dispersal ability through flight was similar throughout their time on a particular island, then the ability of damselflies to disperse through flight could potentially limit the influence of allopatry on speciation.

**Figure 6:**
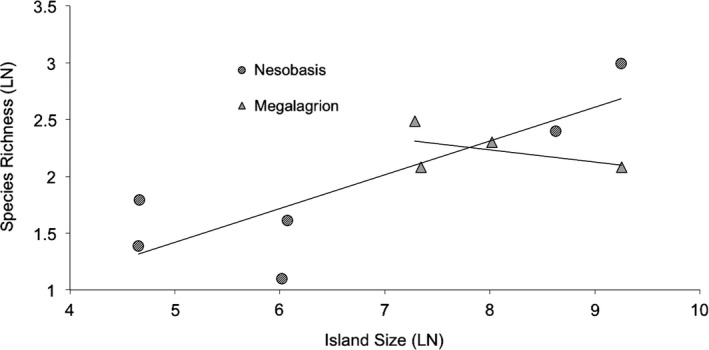
Species richness versus island area for *Nesobasis* and *Megalagrion*. Data from Jordan et al. (2003, 2005), Van Gossum et al. (2008)

As mentioned previously, the other well‐known large radiation of island damselflies is the genus *Megalagrion*, in Hawaii. In *Megalagrion*, speciation appears to have occurred through a combination of inter‐island dispersal events, followed by within‐island speciation through diversification in larval habitats from streams into seeps and plant leaf axils (Jordan et al., 2003, 2005; Polhemus, 1997). A comparison of species richness versus island size for these two genera show marked differences: For *Nesobasis,* we see a trend of increasing species richness with increasing island size, but in *Megalagrion* we do not (Figure 6). It has been suggested that for *Megalagrion*, island age, rather that island size, is associated with greater species richness (Jordan et al., 2003); in Fiji, the largest islands are the oldest, different from the pattern seen in Hawaii.

We are left to conclude that reproductive isolation, possibly paired with allopatry, has driven speciation in *Nesobasis* and *Melanesobasis*, although this still leaves the question of why these mechanisms have resulted in such a large number of species, compared to other island damselfly groups. Another intriguing possibility has been identified: In preliminary analysis, it has been found that 16 of 23 *Nesobasis* species tested were infected with *Wolbachia* bacterial endosymbionts (S. Charlat, personal communication), with infection rates as high as 90% is some population samples. These intracellular parasites are common in a variety of arthropod orders and have been identified to have significant effects on host mating either through the skewing of host sex ratios (through killing or feminization of male hosts) or through induced cytoplasmic incompatibility (CI) within hosts (Brucker & Bordenstein, 2012; Rokas, 2000; Telschow, Hammerstein, & Werren, 2005). We have previously shown that a number of *Nesobasis* species do demonstrate female‐biased sex ratios at oviposition sites (Van Gossum et al., 2008); these species do not appear as part of a single clade in our phylogeny as we predicted, but are found throughout the tree. Also, a direct link between sex ratio skew and *Wolbachia* infection has not been found in these damselflies, as a number of species with sex ratios approaching 1:1 are infected with *Wolbachia*. Sampling of larvae from three *Nesobasis* species (*N. heteroneura*,* N. erythrops* and *N. rufostigma*) showed 1:1 sex ratios at adult emergence, although males of *N. rufostigma* showed earlier mortality than *N. rufostigma* females, and the males and females of the other two species (C. D. Beatty, T. N. Sherratt, H. Van Gossum, unpublished data). The possibility that CI could have influenced rates of speciation in this group is a promising line of research. It should be noted that in including a nuclear sequence (ITS) among our markers used for phylogenetic analysis, we hope to assuage the concern that hybridization events (Schmidt & Sperling, 2008; Shaw, 2002) or gene transfers from intracellular endosymbionts such as *Wolbachia* (Hurst & Jiggins, 2005; Narita, Nomura, Kato, & Fukatsu, 2006; Whitworth, Dawson, Magalon, & Baudry, 2007) may serve to mask the true evolution of the group brought on by analysis of mitochondrial sequences (COI and 12S DNA).

Fiji is host to a number of groups showing high levels of diversity (Evenhuis & Bickel, 2005; Monaghan, Balke, Pons, & Vogler, 2006; Sarnat & Economo, 2012); *Nesobasis* and *Melanesobasis* have been shown to be exemplars of this trend. While our understanding of the forces behind the radiation of these island damselfly genera is incomplete, we have made inroads here into mapping out the patterns of that diversity, and the relationships between species and islands. In future work, we intend to further explore these intriguing island damselflies.

## DATA ACCESSIBILITY

All data reported in the current paper are available in the Supplementary material, and on GenBank.

## CONFLICT OF INTEREST

None declared.

## AUTHOR CONTRIBUTIONS

C.D.B., T.W.D., H.V.G., and T.N.S conceived the ideas; C.D.B., H.V.G., T.N.S, and J.H.S. performed field work and contributed specimens; T.W.D. provided taxonomic identifications; S.K., A.R., and J.H.S. collected the molecular data; S.K. A.R., M.S.H., and J.H.S analyzed the data; C.D.B., M.S.H., and J.H.S. led the writing; M.S.H. contributed to production of graphics.
