# Supplementary material for: Biogeography and systematics of endemic island damselflies: The Nesobasis and Melanesobasis (Odonata: Zygoptera) of Fiji
Source: Ecol Evol. 2017 Aug 18;7(17):7117–29. doi: 10.1002/ece3.3175 (PMC5587492; doi:10.1002/ece3.3175)

*Ecology and Evolution*

**SUPPORTING INFORMATION**

**Biogeography and systematics of endemic island damselflies: the *Nesobasis* and *Melanesobasis* (Odonata: Coenagrionidae) of Fiji**

Christopher D. Beatty, Melissa Sánchez Herrera, Jeffrey H. Skevington, Arash Rashed, Hans Van Gossum, Scott Kelso and Thomas N. Sherratt

**Figure S1** Images of several adults of the damselfly genera *Nesobasis* and *Melanesobasis*. a) *Nesobasis heteroneura* **♂,** Viti Levu b) *N. erythrops* **♂,** Viti Levu c) Tandem (**♂,♀)**  of *N. anguillicolis* ovipositing, Viti Levu d) *N. rufostigma* **♀**, Viti Levu e) *N. brachycerca* **♂,** Vanua Levu f) *N. sp. nov. 8* **♂,** Vanua Levu g) *Melanesobasis flavilabris* **♂,** Viti Levu h) *M. corniculata* **♂,** Viti Levu. Photos courtesy of Hans Van Gossum and Adolfo Cordero Rivera.


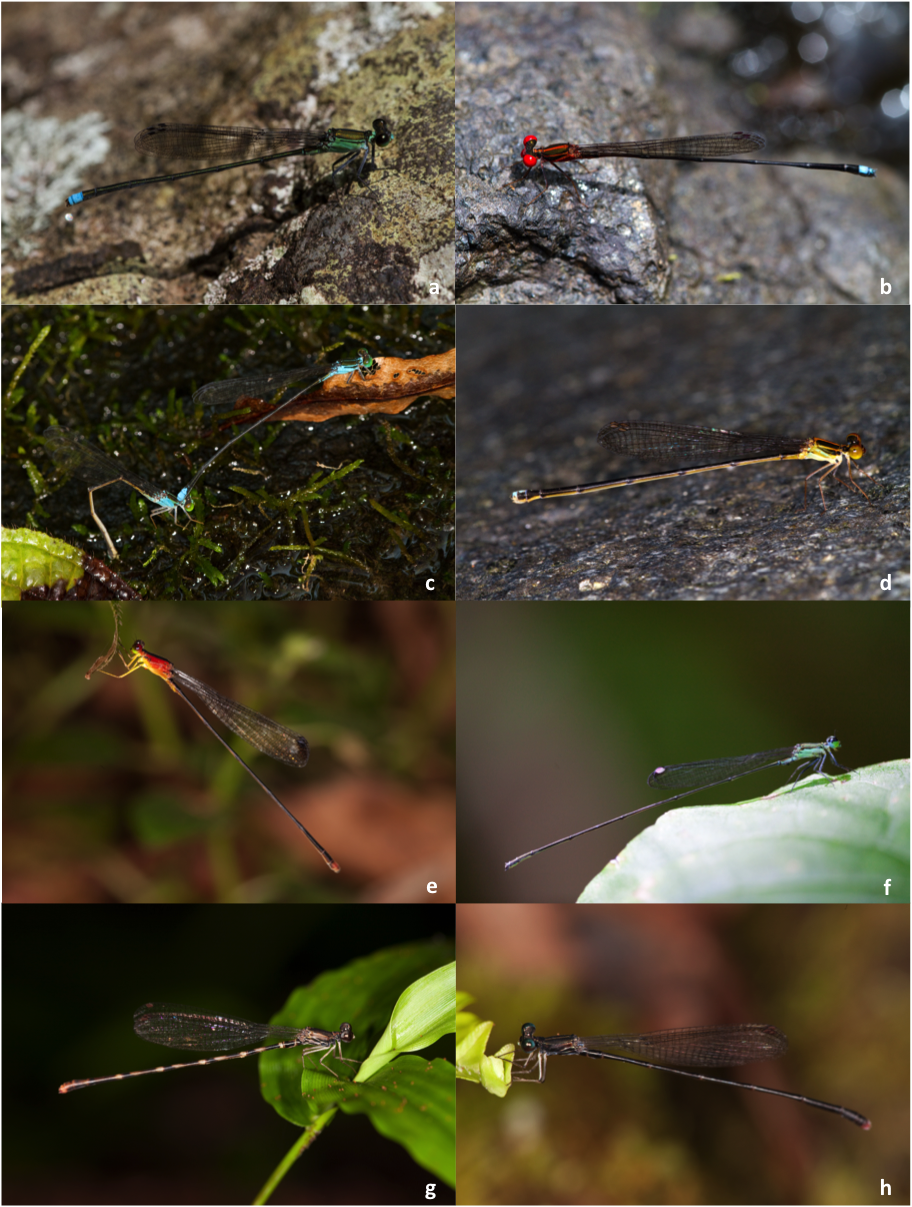


**Figure S2** Results of independent analyses of COI and 12S


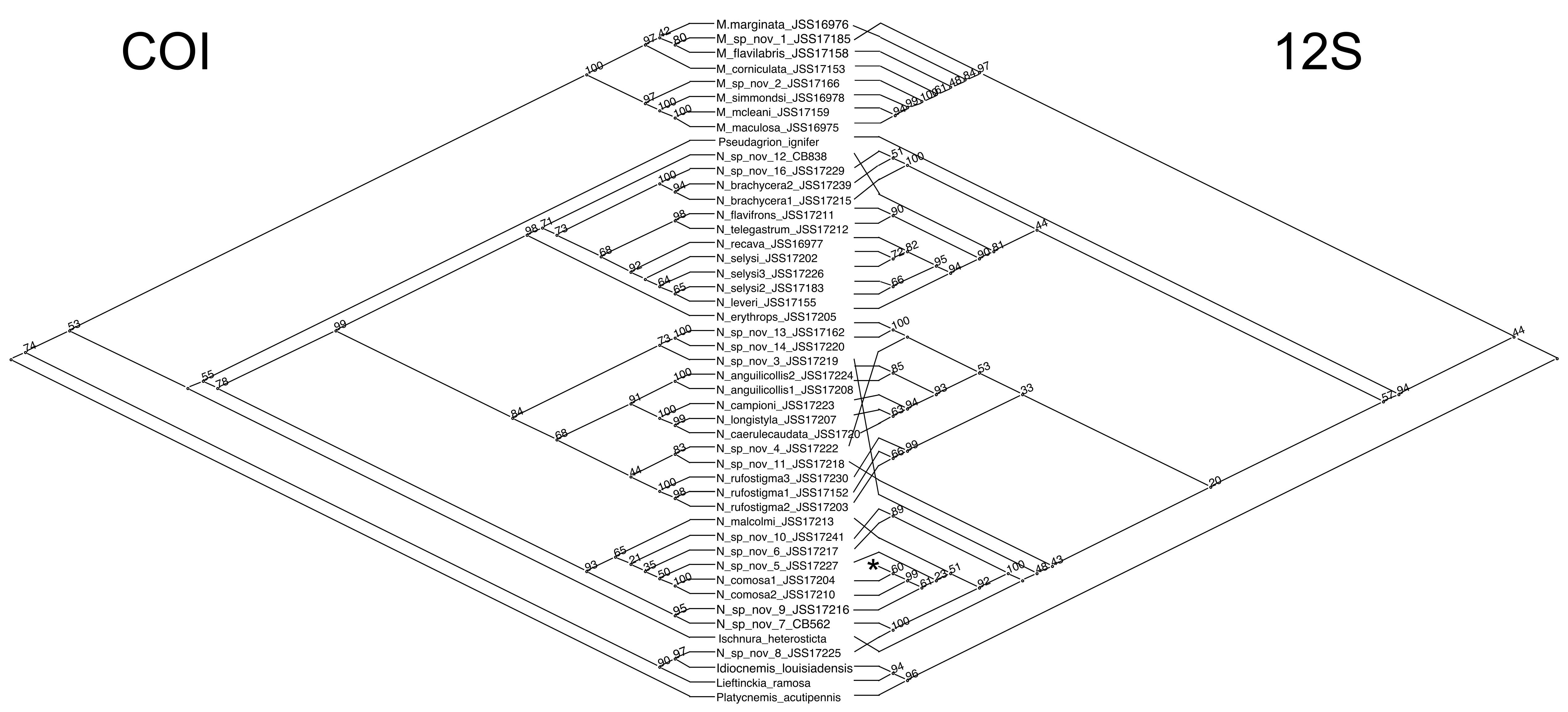


**Figure S3** Results of independent analyses of ITS


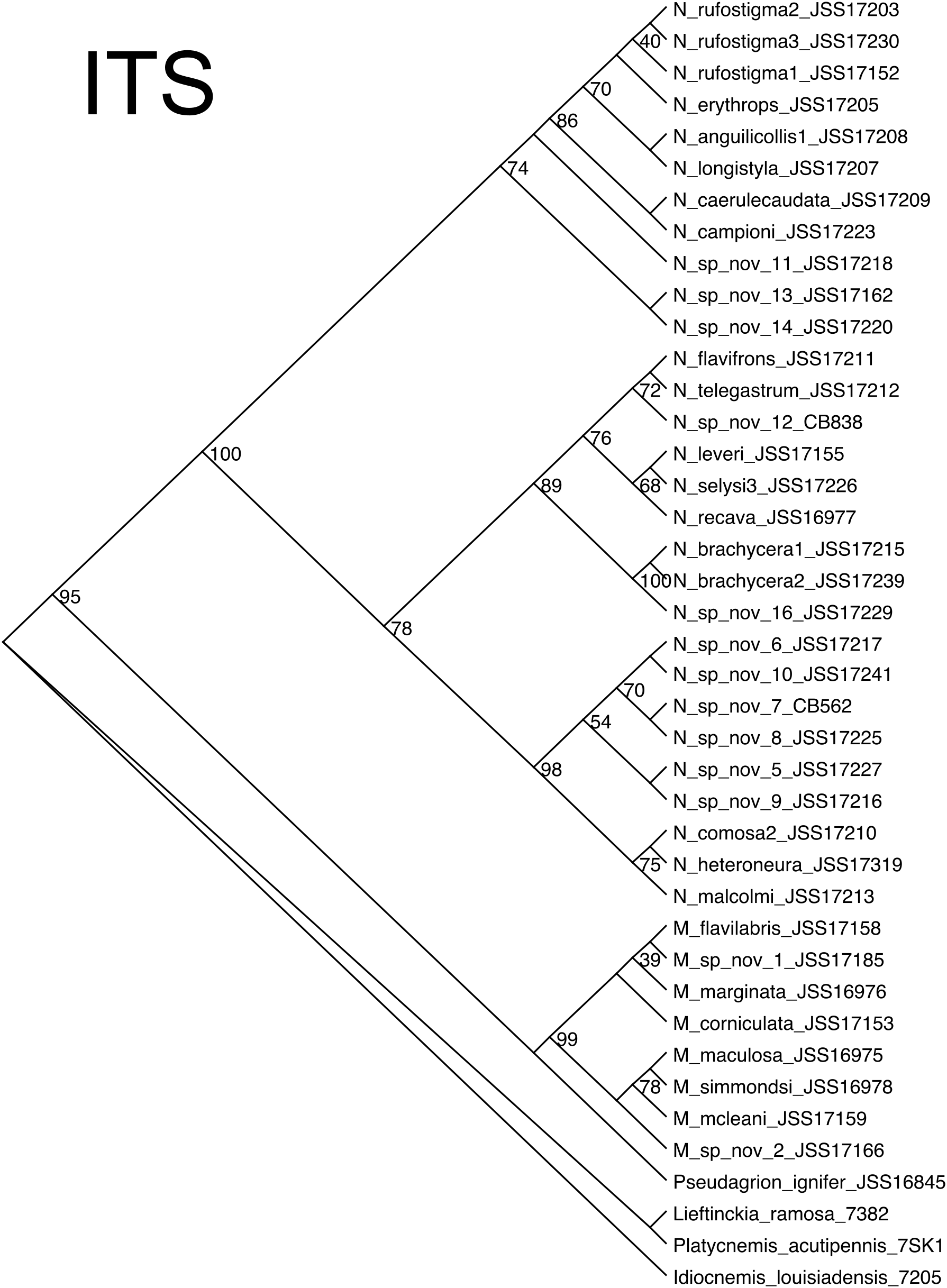

Supplement: Supplementary file 1 [file ECE3-7-7117-s001.docx]
