# Supplementary material for: Biogeography and systematics of endemic island damselflies: The Nesobasis and Melanesobasis (Odonata: Zygoptera) of Fiji
Source: Ecol Evol. 2017 Aug 18;7(17):7117–29. doi: 10.1002/ece3.3175 (PMC5587492; doi:10.1002/ece3.3175)
Supplement: Supplementary file 2 [file ECE3-7-7117-s002.docx]

*Ecology and Evolution*

**SUPPORTING INFORMATION 2**

**Biogeography and systematics of endemic island damselflies: the *Nesobasis* and *Melanesobasis* (Odonata: Coenagrionidae) of Fiji**

Christopher D. Beatty, Melissa Sánchez Herrera, Jeffrey H. Skevington, Arash Rashed, Hans Van Gossum, Scott Kelso and Thomas N. Sherratt

**Table S1** Taxa included in our phylogenetic analysis of the genera *Nesobasis* and *Melanesobasis*, with details on their collection localities. Sequences for 5 outgroup specimens, 35 specimens from the genus *Nesobasis* and 8 specimens from the genus *Melanesobasis* were included.

| **Genus** | **Specific Epithet** | **Specimen** | **Country** | **State** | **Location** | **Latitude** | **Longitude** | **Date** | **Collector** | **Sex** | **Deposition** |
| --- | --- | --- | --- | --- | --- | --- | --- | --- | --- | --- | --- |
| *Ischnura* | *heterosticta* | JSS#16844 | Australia | Queensland | Stanthorpe | 28.665 S | 151.0406 E | 3.i.2000 | JS, AS, MM | ? | CNC |
| *Melanesobasis* | *corniculata corniculata* | JSS#17153 | Fiji | Viti Levu | Logging road to Waivudawa | 18.0736 S | 178.3633 E | 19.i.2006 | JS | M | CNC |
| *Melanesobasis* | *corniculata marginata* | JSS#16976 | Fiji | Taveuni | Mount Devo Peak | 16.8433 S | 179.9658 W | 24.i.2006 | JS | F | CNC |
| *Melanesobasis* | *flavilabris* | JSS#17158 | Fiji | Viti Levu | Colo-i-Suva, Wasili Forest Park Trail | 18.05 S | 178.45 E | 19.i.2006 | JS | M | CNC |
| *Melanesobasis* | *maculosa* | JSS#16975 | Fiji | Viti Levu | Ocean Pacific Resort, Forest Trail | 18.17 S | 178.2569 E | 4.ii.2006 | JS | M | CNC |
| *Melanesobasis* | *mcleani* | JSS#17159 | Fiji | Viti Levu | Colo-i-Suva, Wasili Forest Park Trail | 18.05 S | 178.45 E | 19.i.2006 | JS | M | CNC |
| *Melanesobasis* | *simmondsi* | JSS#16978 | Fiji | Kadavu | Moanakaka Bird Sanctuary | 19.0692 S | 178.1272 E | 21.i.2006 | JS | F | CNC |
| *Melanesobasis* | *sp. nov. 1* | JSS#17185 | Fiji | Kadavu | Namalata Creek | 19.0319 S | 178.1794 E | 21.i.2006 | JS | M | CNC |
| *Melanesobasis* | *sp. nov. 2* | JSS#17166 | Fiji | Taveuni | Bouma Track Upper Falls | 16.83 S | 79.8897 W | 30.i.2006 | JS, MM | M | NDPC |
| *Nesobasis* | *anguilicollis* | JSS#17208 | Fiji | Viti Levu | Sabeto River | 17.7303 S | 177.5444 E | 22.viii.2005 | CB, HVG | M | CNC |
| *Nesobasis* | *anguilicollis* | JSS#17224 | Fiji | Viti Levu | Small stream on road to Abaca | 17.6664 S | 177.5275 E | 23.ix.2005 | CB, HVG | M | CNC |
| *Nesobasis* | *brachycera* | JSS#17215 | Fiji | Vanua Levu | Niuwauvudi Creek | 16.6353 S | 179.7531 E | 17.ix.2005 | CB, HVG | M | CNC |
| *Nesobasis* | *brachycera* | JSS#17239 | Fiji | Koro | Buretini Creek | 17.2636 S | 179.3681 E | 7.ix.2006 | CB, KH | M | CNC |
| *Nesobasis* | *caerulecaudata* | JSS#17209 | Fiji | Viti Levu | Stream crossing Vaturu Dam Road | 17.4364 S | 177.6097 E | 22.viii.2005 | CB, HVG | M | CNC |
| *Nesobasis* | *campioni* | JSS#17223 | Fiji | Viti Levu | Waterfall above Wainikovu Trib. | 18.1122 S | 178.1869 E | 22.viii.2005 | CB, HVG | F | CNC |
| *Nesobasis* | *comosa* | JSS#17204 | Fiji | Viti Levu | Wainikovu Creek | 18.1064 S | 178.1803 E | 11.viii.2008 | CB, HVG | M | CNC |
| *Nesobasis* | *comosa* | JSS#17210 | Fiji | Viti Levu | Stream crossing Vaturu Dam Road | 17.7706 S | 177.6097 E | 22.viii.2005 | CB, HVG | M | CNC |
| *Nesobasis* | *erythrops* | JSS#17205 | Fiji | Viti Levu | Korowaiwai Creek | 17.6031 S | 177.9472 E | 14.viii.2005 | CB, HVG | M | CNC |
| *Nesobasis* | *flavifrons* | JSS#17211 | Fiji | Viti Levu | Stream crossing Vaturu Dam Road | 17.7706 S | 177.6097 E | 22.viii.2005 | CB, HVG | M | CNC |
| *Nesobasis* | *heteroneura* | JSS17319 | Fiji | Viti Levu | Waikubukubu River | 17.5472 S | 177.9436 E | 12.viii.2006 | CB, HVG | F | CNC |
| *Nesobasis* | *leveri* | JSS#17155 | Fiji | Viti Levu | Logging road to Waivudawa | 18.0736 S | 178.3633 E | 19.i.2006 | JS | M | CNC |
| *Nesobasis* | *longistyla* | JSS#17207 | Fiji | Viti Levu | Stream crossing Vaturu Dam Road | 17.7706 S | 177.1097 E | 22.viii.2005 | CB, HVG | M | CNC |
| *Nesobasis* | *malcomi* | JSS#17213 | Fiji | Viti Levu | National Park near Abaca Village | 17.6686 S | 177.5419 E | 23.viii.2005 | CB, HVG | F | CNC |
| *Nesobasis* | *recava* | JSS#16977 | Fiji | Kadavu | Moanakaka Bird Sanctuary | 19.0692 S | 178.1272 E | 21.i.2006 | JS | M | CNC |
| *Nesobasis* | *rufostigma* | JSS#17152 | Fiji | Kadavu | Moanakaka Bird Sanctuary | 19.0692 S | 178.1272 E | 21.i.2006 | JS | F | CNC |
| *Nesobasis* | *rufostigma* | JSS#17203 | Fiji | Viti Levu | Vago Creek | 18.0814 S | 178.4428 E | 11.viii.2005 | CB, HVG | F | CNC |
| *Nesobasis* | *rufostigma* | JSS#17230 | Fiji | Koro | Buretini Creek | 17.2636 S | 179.3681 E | 7.ix.2006 | CB, HVG | F | CNC |
| *Nesobasis* | *selysi* | JSS#17202 | Fiji | Viti Levu | Small tributary of Qualiwana Creek | 17.6075 S | 177.9889 E | 14.viii.2005 | CB, HVG | M | CNC |
| *Nesobasis* | *selysi* | JSS#17183 | Fiji | Viti Levu | Vereni Falls near Lautoka | 17.6833 S | 177.5167 E | 17.i.2006 | JS | F | CNC |
| *Nesobasis* | *selysi* | JSS#17226 | Fiji | Viti Levu | Korowaiwai Creek | 17.6031 S | 177.9472 E | 14.viii.2005 | CB, HVG | F | CNC |
| *Nesobasis* | *telegastrum* | JSS#17212 | Fiji | Viti Levu | Small stream on road to Abaca | 17.6664 S | 177.5275 E | 23.viii.2005 | CB, HVG | M | CNC |
| *Nesobasis* | *sp. nov. 3* | JSS#17219 | Fiji | Vanua Levu | Lomaloma Falls | 16.6231 S | 179.1667 E | 18.ix.2005 | CB, HVG | F | CNC |
| *Nesobasis* | *sp. nov. 4* | JSS#17222 | Fiji | Vanua Levu | Sauvuqoro Creek, in Waisali Nature Preserve | 16.6417 S | 179.225 E | 20.ix.2005 | CB, HVG | M | CNC |
| *Nesobasis* | *sp. nov. 5* | JSS#17227 | Fiji | Viti Levu | Wainivesi Creek | 14.6686 S | 177.9378 E | 8.ix.2005 | CB, HVG | F | CNC |
| *Nesobasis* | *sp. nov. 6* | JSS#17217 | Fiji | Vanua Levu | Lomaloma Falls | 16.6231 S | 179.1667 E | 18.ix.2005 | CB, HVG | M | CNC |
| *Nesobasis* | *sp. nov. 7* | CB562 | Fiji | Vanua Levu | Volivoli Creek | 16.6292 S | 179.1681 E | 18.ix.2005 | CB, HVG | F | Lost |
| *Nesobasis* | *sp. nov. 8* | JSS#17225 | Fiji | Vanua Levu | Stream on road above Korotesere | 16.5131 S | 179.545 E | 27.ix.2006 | CB, HVG | M | CNC |
| *Nesobasis* | *sp. nov. 9* | JSS#17216 | Fiji | Vanua Levu | Lomaloma Falls | 18.1064 S | 179.1667 E | 18.ix.2005 | CB, HVG | F | CNC |
| *Nesobasis* | *sp. nov. 10* | JSS17241 | Fiji | Koro | Large stream on E side of Koro | 17.3303 S | 179.4319 E | 8.ix.2006 | CB, KH | M | CNC |
| *Nesobasis* | *sp. nov. 11* | JSS#17218 | Fiji | Vanua Levu | Small stream above Saivou | 16.6136 S | 179.1519 E | 18.ix.2005 | CB, HVG | M | CNC |
| *Nesobasis* | *sp. nov. 12* | CB838 | Fiji | Vanua Levu | Sauvuqoro Creek | 16.6417 S | 179.225 E | 20.ix.2005 | CB, HVG | M | Lost |
| *Nesobasis* | *sp. nov. 13* | JSS#17162 | Fiji | Taveuni | Bouma Track Upper Falls | 16.83 S | 179.8897 W | 30.i.2006 | JS, MM | M | CNC |
| *Nesobasis* | *sp. nov. 14* | JSS#17220 | Fiji | Vanua Levu | Raviravi Creek | 16.6072 S | 179.1478 E | 19.ix.2005 | CB, HVG | M | CNC |
| *Nesobasis* | *sp. nov. 16* | JSS#17229 | Fiji | Taveuni | Navaka Creek at Tavoro Falls, Bouma | 16.8167 S | 179.8667 W | 14.viii.2005 | DP, MT | ? | CNC |
| *Pseudagrion* | *ignifer* | JSS#16845 | Australia | Queensland | Brisbane Forest Park, Northbrook Creek | 27.3056 S | 152.6903 E | 19.iii.2000 | JS | M | CNC |
|  |  |  |  |  |  |  |  |  |  |  |  |

Abbreviations: CB – Chris Beatty, MM – Michael Mathieson, DP – D. A. Polhemus, AS – Angela Skevington, JS – Jeff Skevington, MT – M. Tokota’a, HVG – Hans Van Gossum, KH – Katie Harding; M – male, F – female; CNC – Canadian National Collection of Insects, Arachnids and Nematodes, NDPC – Nick Donnelly Personal Collection.

**Table S2** Primers used in phylogenetic analysis.

**Table S3** GenBank Accession numbers for sequences used in our phylogenetic analysis. NA indicates sequences unavailable for an individual specimen.

|  | Sequence | | |  | |  | |  |
| --- | --- | --- | --- | --- | --- | --- | --- | --- |
| SPECIMEN | | ITS | COIPJ | | cox1 | | 12s | |
| IheterostictaJSS16844 | | NA | FJ812773 | | FJ812817 | | FJ812728 | |
| PigniferJSS16845 | | FJ812861 | FJ812774 | | FJ812818 | | FJ812729 | |
| McorniculataJSS17153 | | FJ812862 | FJ812775 | | FJ812819 | | FJ812730 | |
| MflavilabrisJSS17158 | | FJ812863 | FJ812776 | | FJ812820 | | FJ812731 | |
| MmaculosaJSS16975 | | FJ812864 | FJ812777 | | FJ812821 | | FJ812732 | |
| MmarginataJSS16976 | | FJ812865 | FJ812778 | | FJ812822 | | FJ812733 | |
| MmcleaniJSS17159 | | FJ812866 | FJ812779 | | FJ812823 | | FJ812734 | |
| MsimmondsiJSS16978 | | FJ812867 | FJ812780 | | FJ812824 | | FJ812735 | |
| Mspnov1JSS17185 | | FJ812869 | FJ812782 | | FJ812826 | | FJ812737 | |
| Mspnov2JSS17166 | | FJ812868 | FJ812781 | | FJ812825 | | FJ812736 | |
| NangulicollisJSS17208 | | FJ812871 | FJ812784 | | FJ812828 | | FJ812739 | |
| NangulicollisJSS17224 | | NA | FJ812785 | | FJ812829 | | FJ812740 | |
| NbrachycercaJSS17215 | | FJ812872 | FJ812787 | | FJ812831 | | FJ812742 | |
| NbrachycercaJSS17239 | | FJ812874 | FJ812789 | | FJ812833 | | FJ812744 | |
| NcaerulecaudataJSS17209 | | FJ812875 | FJ812790 | | FJ812834 | | FJ812745 | |
| NcampioniJSS17223 | | FJ812876 | FJ812791 | | FJ812835 | | FJ812746 | |
| NcomosaJSS17204 | | NA | FJ812792 | | FJ812836 | | FJ812747 | |
| NcomosaJSS17210 | | FJ812877 | FJ812793 | | FJ812837 | | FJ812748 | |
| NerythropsJSS17205 | | FJ812879 | FJ812795 | | FJ812839 | | FJ812750 | |
| NflavifronsJSS17211 | | FJ812880 | FJ812796 | | FJ812840 | | FJ812751 | |
| NheteroneuraJSS17319 | | FJ812882 | NA | | FJ812842 | | FJ812753 | |
| NleveriJSS17155 | | FJ812883 | FJ812798 | | FJ812843 | | FJ812754 | |
| Nlongistyla1JSS17207 | | FJ812884 | FJ812799 | | FJ812844 | | FJ812755 | |
| NmalcolmiJSS17213 | | FJ812886 | FJ812801 | | FJ812846 | | FJ812757 | |
| NrecavaJSS16977 | | FJ812890 | FJ812806 | | NA | | FJ812762 | |
| NrufostigmaJSS17152 | | FJ812892 | FJ812808 | | FJ812852 | | FJ812764 | |
| NrufostigmaJSS17203 | | FJ812893 | FJ812809 | | FJ812853 | | FJ812765 | |
| NrufostigmaJSS17230 | | FJ812894 | FJ812810 | | FJ812854 | | FJ812766 | |
| NselysiJSS17183 | | NA | FJ812802 | | FJ812847 | | FJ812758 | |
| NselysiJSS17202 | | NA | FJ812811 | | FJ812855 | | FJ812767 | |
| NselysiJSS17226 | | FJ812887 | FJ812803 | | FJ812848 | | FJ812759 | |
| NtelegastrumJSS17212 | | FJ812895 | FJ812812 | | FJ812856 | | FJ812768 | |
| Nspnov3JSS17219 | | NA | FJ812786 | | FJ812830 | | FJ812741 | |
| Nspnov4JSS17222 | | NA | FJ812813 | | FJ812857 | | FJ812769 | |
| Nspnov5JSS17227 | | FJ812885 | FJ812800 | | FJ812845 | | FJ812756 | |
| Nspnov6JSS17217 | | FJ812888 | FJ812804 | | FJ812849 | | FJ812760 | |
| Nspnov7CB562 | | FJ812889 | FJ812805 | | FJ812850 | | FJ812761 | |
| Nspnov8JSS17225 | | FJ812898 | FJ812816 | | FJ812860 | | FJ812772 | |
| Nspnov9JSS17216 | | FJ812878 | FJ812794 | | FJ812838 | | FJ812749 | |
| Nspnov11JSS17218 | | FJ812870 | FJ812783 | | FJ812827 | | FJ812738 | |
| Nspnov12CB838 | | FJ812891 | FJ812807 | | FJ812851 | | FJ812763 | |
| Nspnov13JSS17162 | | FJ812896 | FJ812814 | | FJ812858 | | FJ812770 | |
| Nspnov14JSS17220 | | FJ812897 | FJ812815 | | FJ812859 | | FJ812771 | |
| Nspnov16JSS17229 | | FJ812873 | FJ812788 | | FJ812832 | | FJ812743 | |

**Table S4** Taxonomical and locality information of fossils; and biogeographical features used for the Divergence Time Analyses node calibration. Includes the prior distributions.

| Node | Fossil and Deposit Locality | Type Specimen/PaleoDB | Prior distribution |
| --- | --- | --- | --- |
| NA | Caloptera, Dysagrionidae † , *Eucarchistigma marialuiseae † and E. peterknobli †*, Early Cretaceous (Bechly 2007, 2010) | AMNH44204/  PaleoDB 122919,122922 | Used as max bound in the uniform prior distributions max = 150 |
|  | Caloptera, Hemiphlebiidae, *Electrohemiphlebia barucheli †,* Early Cretaceous-Late Jurassic (Lak *et al* 2009) | ARC 372.1/unknown |  |
| Outgroup | Coenagrionidae, *Ischnura velteni,* Miocene, Burdigalian (Bechly, 2000) | PaleoDB  SMNS Do-5687 | Uniform; max=150; std=13 |
| Ingroup/ *Nesobasis* and *Melanesobasis* | Viti Levu uplift formation , ~28 to 12Ma (Neall and Trewick 2008) | NA | LogNormal, Mean=12; std=0.3 |
| Ingroup/  *N. crassa*, sp.nov 7 and 8 | Vana Levu uplift formation , ~7Ma (Neall and Trewick 2008) | NA | LogNormal, Mean=7;  std=2 |

G. Bechly. 2000. A new fossil damselfly species (Insecta: Odonata: Zygoptera: Coenagrionidae: Ischnurinae) from Dominican Amber. *Stuttgarter Beiträge zur Naturkunde Serie B (Geologie und Paläontologie)* **299**:1-9

G. Bechly. 2007. Odonata: damselflies and dragonflies. *The Crato Fossil Beds of Brazil: Window into an Ancient World* 184-222

G. Bechly. 2010. Additions to the fossil dragonfly fauna from the Lower Cretaceous Crato Formation of Brazil (Insecta: Odonata). *Palaeodiversity* **3(Supplement)**:11-77

M. Lak, G. Fleck, D. Azar, M. S. Engel, H. F. Kaddumi, D. Neraudeau, P. Tafforeau and A. Nel. 2009. Phase contrast X-ray synchrotron microtomography and the oldest damselflies in amber (Odonata: Zygoptera: Hemiphlebiidae). *Zoological Journal of the Linnean Society* **156**:913-923

**Table S5** Output from RASP DEC and S-DEC analyses. At each node in the tree, relative probabilities (RP) for each vicariance scenario are provided. The clades represented at each node are provided in each row; geographic codes are as follows: A = Viti Levu, B = Vana Levu, C = Kadavu, D = Taveuni, E = Ovalau, F = Koro, G = Mainland; numbers in parentheses correspond to numbered nodes in Figure 4?.

| **Node / Route and probability (**p=DEC – S-DEC**)** | **DEC** | | **S-DEC** | |
| --- | --- | --- | --- | --- |
|  | **Ancestral Area** | **RP (%)** | **Ancestral**  **Area** | **RP (%)** |
| Root (95)  AG->AG^G->AG\|G, p=0.1130 – 0.1125 | AG  G  A  BG  CG | 40.76  35.08  11.89  6.72  5.56 | AG  G  A  BG  CG | 40.59  34.30  10.64  5.65  2.39 |
| Outgroups (Platycnemidae, 50)  G->G^G->G\|G , p=0.6901 – 0.7394 | G  AG | 79.2  20.8 | G  AG  A | 80.15  17.85  2 |
| Coenagrionidae (*Melanesobasis, Nesobasis Ischnura* and *Pseudagrion*, 94) | AG  G  A | 35  32.89  32.11 | G  AG  A | 34.58  34.42  31 |
| *Melanesobasis* (93)  A->A^A->AD^A->AD\|A, p=0.6028 – 0.5055 | A | 100 | A  AG  AD | 97.52  1.96  0.51 |
| *M. sp.nov. 2, M. simmondsi, M.maculosa, M.mcleani* (92)  AD->D\|A, p=0.3347 – 0.3335 | AD  A | 60.28  39.72 | AD  A  AC | 52.22  47.33  0.45 |
| *M.mcleani, M. maculosa, M.simmondsi* (91)  A->A^A->AC^A->AC\|A, p=0.5553 – 0.6387 | A  AC | 55.53  44.47 | A  AC | 63.87  36.13 |
| *M.mcleani, M. maculosa* (90)  A->A^A->A\|A, p=1 | A | 100 | A | 100 |
| *M.corniculata, M.flavilabris, M.sp.nov. 1, M.marginata* (89)  A->A^A->ABDCEF^A->ABDCEF\|A, p= 1 – 0.9799 | A | 100 | A  AC | 99.27  0.73 |
| *M.flavilabris, M.sp.nov. 1, M.marginata* (88)  A->A^A->A\|A, p=1 – 0.6550 | A | 100 | A  AC | 98.71  1.29 |
| *M.flavilabris, M.sp.nov. 1* (87)  A->CA->C\|A, p=0.6936 – 0.6636 | A  AC | 69.36  30.64 | A  AC | 66.36  33.64 |
| *Nesobasis, Ischnura, Pseudagrion* (86)  G->G^G->GA^G->G\|AG, p=0.3027 – 0.2905 | G  A  AG | 44.17  30.39  25.44 | G  A  AG | 46.91  28.48  24.62 |
| *Nesobasis, Ischnura* (85)  AG->G\|A, p=0.5634 – 0.4696 | AG  A | 68.52  31.48 | AG  A  BG  G  AB  B | 61.92  28.36  5.33  3.96  0.36  0.88 |
| *Nesobasis* (84)  A->A^A->A\|A, p=0.2952 -0.3382 | A  AB | 82.23  17.77 | A  AB  B | 75.84  20.62  3.53 |
| longistyla, erythrops B, erythops A (83)  A->A^A->AB^A->AB\|A, p=0.6728 – 0.5174 | A  AB | 67.28  32.72 | A  AB  B | 60.84  38.70  0.46 |
| longistyla, erythrops B (82)  AB->B\|A, p=0.7229 – 0.6056 | AB | 100 | AB  B  A  AD | 87.91  10.70  1.28  0.12 |
| *N. sp.nov. 3, N. sp.nov. 13&14, N. sp.nov. 4, N.sp.nov. 11* (81)  B->B^B->BD^B->BD\|B, p=1 – 0.9680 | B | 100 | B  AB | 98.37  1.63 |
| *N. sp.nov. 13&14, N. sp.nov. 4, N.sp.nov. 11* (80)  B->B^B->B\|B, p=0.7722 – 0.7740 | B | 100 | B  BD | 98.40  1.60 |
| *N. sp.nov. 13&14, N. sp.nov. 4* (79)  B->B^B^D->BD^B^D->BD\|BD, p=0.6581- 0.6923 | B  BD | 77.22  22.78 | B  BD | 78.66  21.34 |
| *N.rufostigma, N. anguilicollis, N.campioni, N.caerulecaudata, N.longistyla* (77)  A->A^A->A\|A, p=0.5121 – 0.4761 | A  AB | 72.29  27.71 | A  AB  B | 70.03  29.72  0.25 |
| *N. anguilicollis, N.campioni, N.caerulecaudata, N.longistyla* (76)  A->A^A->A\|A, p=1 | A | 100 | A | 100 |
| *N.campioni, N.caerulecaudata, N.longistyla* (75)  A->A^A->A\|A, p=1 – 0.9511 | A | 100 | A | 100 |
| *N.caerulecaudata, N.longistyla* (74)  A->A^A->AC^A->A\|AC, p=1 – 0.9511 | A | 100 | A  AC | 95.11  4.89 |
| erythrops A (70)  A->A^A->A\|A, p=1 – 0.9062 | A | 100 | A  AB | 96.74  3.26 |
| *N.sp.nov. 12, N.telegastrum, N.flavifrons, N.recava, N.selysi, N.leveri, N.recava, N.sp.nov. 16, N.brachycera* (69)  A->A^A->AE^A->A\|AE, p=0.7433 – 0.6187 | A | 100 | A  AB | 93.67  6.33 |
| *N.sp.nov. 12, N.telegastrum, N.flavifrons, N.recava, N.selysi, N.leveri* (68)  A->BA->B\|A, p=0.7433 – 0.6970 | A  AB | 74.33  25.67 | A  AB | 71.98  28.02 |
| *N.telegastrum, N.flavifrons, N.recava, N.selysi, N.leveri* (67)  A->A^A->AC^A->A\|AC, p=0.6953– 0.6467 | A | 100 | A  AC | 96.83  3.17 |
| *N.telegastrum, N.flavifrons* (66)  A->A^A->A\|A, p=1 | A | 100 | A | 100 |
| *N.selysi, N.leveri N.recava* (65)  AC->CAE->C\|AE, p=0.4298 – 0.3685 | AC  A | 69.53  30.47 | AC  A  AE | 66.79  33.14  0.08 |
| *N.selysi, N.leveri* (64)  AE->AE^A->A\|AE, p=0.4471 – 0.3277 | AE  A | 61.82  38.18 | AE  A | 55.18  44.82 |
| *N.sp.nov. 16, N.brachycera* (61)  AE->AE^A->AE\|A, p=0.5563 – 0.5162 | AE | 100 | AE  A | 91.76  8.24 |
| comosa (59)  A->A^A->AB^A->A\|AB, p=0.2797 – 0.4794 | A  AB | 53.35  46.65 | AB  A  B | 73.30  26.09  0.61 |
| *N.malcomi, N. sp.nov. 9, N.sp nov 8, N. sp nov 7, N. sp. nov. 10, N. sp.nov. 6, N.comosa, N.heteroneura* (58)  AB->AB^A->AB\|A, p=0.1545 – 0.2404 | AB  A | 52.42  47.58 | AB  A  B | 65.40  33.12  1.48 |
| *N.malcomi, N.sp.nov. 9, N.sp nov 8, N. sp nov 7* (57)  AB->A\|B, p=0.5310 – 0.6755 | AB  B  A | 64.04  19.36  16.6 | AB  B  A | 72.08  15.27  12.65 |
| *N.sp.nov. 9, N.sp nov 8, N. sp nov 7* (56)  B->B^B->B\|B, p=0.8292-0.9372 | B  AB | 82.92  17.08 | B  AB | 93.72  6.28 |
| *N. sp.nov. 10, N. sp.nov. 6, N.comosa, N.heteroneura* (54)  A->BA->B\|A, p=0.2251-0.3598 | A  AB  B | 46.03  40.78  13.19 | AB  A  B | 51  39.71  9.28 |
| *N. sp. nov. 10, N. sp.nov. 6* (53)  B->B^B->BDF^B->B\|BDF, p=0.4891-0.71 | B  AB  A | 48.91  31.60  19.49 | B  AB  A | 70.55  20.68  8.77 |
| *N.comosa, N.heteroneura* (52)  A->A^A->A\|A, p=1 | A | 100 | A | 100 |
